# Increasing importance of European lineages in seeding the hepatitis C virus subtype 1a epidemic in Spain

**DOI:** 10.2807/1560-7917.ES.2019.24.9.1800227

**Published:** 2019-02-28

**Authors:** Ana Belen Pérez, Bram Vrancken, Natalia Chueca, Antonio Aguilera, Gabriel Reina, Miguel García-del Toro, Francisco Vera, Miguel Angel Von Wichman, Juan Ignacio Arenas, Francisco Téllez, Juan A Pineda, Mohamed Omar, Enrique Bernal, Antonio Rivero-Juárez, Elisa Fernández-Fuertes, Alberto de la Iglesia, Juan Manuel Pascasio, Philippe Lemey, Féderico Garcia, Lize Cuypers

**Affiliations:** 1 Department of Microbiology, Institute of Bio Sanitary Research (IBIS), AIDS Research Network, University Hospital of Granada, Granada, Spain; 2 These authors contributed equally to the article; 3 KU Leuven, Department of Microbiology and Immunology, Rega Institute for Medical Research, Laboratory of Evolutionary and Computational Virology, Leuven, Belgium; 4 Department of Microbiology, University Hospital of Santiago, Santiago de Compostela, Spain; 5 Department of Microbiology, University Hospital of Navarra, Institute for Health Research (IdisNA), Pamplona, Spain; 6 Unit of Infectious Diseases, General Hospital of Valencia, Valencia,; 7 Unit of Infectious Diseases, Internal Medicine, General Hospital of Rosell, Cartagena, Murcia, Spain; 8 Unit of Infectious Diseases, Hospital Universitario de San Sebastian, San Sebastian, Spain; 9 Unit of Infectious Diseases and Microbiology, University Hospital of Puerto Real, Cádiz, Spain; 10 Unit of Infectious Diseases, University Hospital of Valme, Sevilla, Spain (J.A. Pineda); 11 University Hospital of Jaen, Jaen, Spain; 12 Unit of Infectious Diseases, General University Hospital, Murcia, Spain; 13 Unit of Infectious Diseases, University Hospital Reina Sofía of Córdoba, Maimonides Institute of Biomedical Research of Córdoba, University of Córdoba, Córdoba, Spain; 14 Unit of Tropical Medicine, Hospital of Poniente, El Ejido, Almería, Spain; 15 Department of Microbiology, Hospital Infanta Elena of Huelva, Huelva, Spain; 16 Clinical Management Unit of Digestive Diseases, University Hospital of Virgen del Rocío, Sevilla, Spain; 17 KU Leuven, Department of Microbiology and Immunology, Rega Institute for Medical Research, Laboratory of Clinical and Epidemiological Virology, Leuven, Belgium

**Keywords:** HCV1a, Spain, Europe, North America, phylogeography, public health, hepatitis C virus, public health policy

## Abstract

**Background:**

Reducing the burden of the hepatitis C virus (HCV) requires large-scale deployment of intervention programmes, which can be informed by the dynamic pattern of HCV spread. In Spain, ongoing transmission of HCV is mostly fuelled by people who inject drugs (PWID) infected with subtype 1a (HCV1a).

**Aim:**

Our aim was to map how infections spread within and between populations, which could help formulate more effective intervention programmes to halt the HCV1a epidemic in Spain.

**Methods:**

Epidemiological links between HCV1a viruses from a convenience sample of 283 patients in Spain, mostly PWID, collected between 2014 and 2016, and 1,317, 1,291 and 1,009 samples collected abroad between 1989 and 2016 were reconstructed using sequences covering the NS3, NS5A and NS5B genes. To efficiently do so, fast maximum likelihood-based tree estimation was coupled to a flexible Bayesian discrete phylogeographic inference method.

**Results:**

The transmission network structure of the Spanish HCV1a epidemic was shaped by continuous seeding of HCV1a into Spain, almost exclusively from North America and European countries. The latter became increasingly relevant and have dominated in recent times. Export from Spain to other countries in Europe was also strongly supported, although Spain was a net sink for European HCV1a lineages. Spatial reconstructions showed that the epidemic in Spain is diffuse, without large, dominant within-country networks.

**Conclusion:**

To boost the effectiveness of local intervention efforts, concerted supra-national strategies to control HCV1a transmission are needed, with a strong focus on the most important drivers of ongoing transmission, i.e. PWID and other high-risk populations.

## Introduction

An estimated 70 million people are currently infected with the hepatitis C virus (HCV) and about 400.000 die each year from HCV-related chronic liver diseases. Highly effective antiviral regimens have become available but, as up to 80% of infected patients remain undiagnosed [1], scaling up treatment alone will not suffice to eradicate the virus on a global scale. Prevention of new infections remains a primary objective, especially because the number of new infections has been rising since 2010 [2]. While HCV subtypes often have a shared geographical distribution or comparable prevalence rates among risk groups in the same location, this does not imply that their distribution is necessarily shaped by similar transmission histories [3]. Because specific sub-epidemic patterns of spread can undermine the success of public health intervention programmes, a case-by-case investigation that considers local characteristics is warranted [4,5].

HCV accrues genetic differences on the same time scale as epidemiological processes develop, making it possible to trace the spread of the virus among individuals from viral genetic information using phylogenetic approaches [6]. A phylogenetic tree depicts the ancestor–descendant relationships between the viruses that infect different individuals through branches that connect the observed data (the tips) and the inferred, unobserved ancestors (the nodes). Contrary to traditional epidemiological methods, coalescent-based phylogenetic reconstructions need only a small number of samples to infer the population dynamics over time. However, small datasets do not provide much resolution on transmission linkage and migration histories, which largely depend on the availability of genetic information of HCV strains circulating in epidemiologically relevant populations [7]. Fortunately, increasingly detailed sampling of the global and Spanish HCV1a epidemic has become available in recent years thanks to expanding sequencing efforts in the context of routine care and drug resistance research [8]. This allows us to bypass the reconstruction of contact networks from patient interviews, which are difficult to interpret because of the chronic nature of HCV infection.

The estimated prevalence of HCV in Spain is similar to other countries in Western Europe and lies between 0.5% and 1% [9]. HCV1a is responsible for a quarter of all HCV infections in Spain [10,11] and reaches up to 40% among people who inject drugs (PWID), the risk group at the core of the current HCV epidemic [10,12]. So far, the Spanish national HCV programme has prioritised universal access to treatment [13], ensuring a large increase in the uptake of antiviral therapy across infected populations. However, it has been estimated that around 60–70% of the Spanish HCV-infected population remains undiagnosed. Recent data indicate that per five patients that are cured, only one patient is newly diagnosed, suggesting that Spain will soon reach a diagnostic burn-out [1]. The need for extended screening efforts with a focus on the populations at highest risk is now being addressed and is complemented by prevention strategies such as needle exchange and opioid substitution programmes [13]. The effectiveness of local and national intervention efforts, however, partly depends on the extent to which transmission is driven by local propagation: when an epidemic is driven by migration, the success of local intervention programmes is co-determined by how well transmission can be interrupted elsewhere [14].

Here, we capitalise on sequence data of the NS3, NS5A and NS5B genes generated in the context of the Group for the Study of Viral Hepatitis (GEHEP) national programme to evaluate the relevance of virus movements for the Spanish HCV1a epidemic.

## Methods

### Newly generated sequence data for the hepatitis C virus from Spain

We generated viral sequences for a convenience sample of 283 HCV1a infected patients who attended one of the 24 specialised clinical centres spread throughout Spain (Supplementary Figure S1) and participated in the GEHEP programme. At the time of sampling, 231 patients were naive for direct acting antiviral (DAA) treatment, while for 52 patients, a viral sequence at time of therapy failure was obtained because no sample was available at baseline. In the context of antiviral drug resistance, genetic sequencing was performed at the San Cecilio Hospital in Granada. Dependent on the composition of the DAA regimen that was administered and on the sample’s volume, one, two or three genetic regions in the HCV genome (NS3, NS5A and NS5B) were targeted using assays developed in house (see Supplement). Overall, 398 amplification and sequencing reactions were successful, and the first available sample from each patient was used in subsequent analyses. Of these, data from all three fragments were available for 37 patients, for another 24 patients, two genomic regions were successfully sequenced and for the remaining 222 patients, one viral genetic sequence was obtained. All sequences generated in this study have been submitted to GenBank (accession numbers: MG738825–MG739222). 

### Dataset compilation

The newly generated sequence data from Spain were complemented with time- and geo-referenced viral genetic data available in GenBank (https://www.ncbi.nlm.nih.gov/) following the methodology detailed by Cuypers et al. [4]. Briefly, the procedure started from the sequences that were unambiguously subtyped as HCV1a. Clonal sequences, strains from non-human hosts and duplicate data were excluded and a multiple sequence alignment was generated using a codon-correct alignment tool [15]. Sequences with stop codons and those covering less than 80% of the new NS3, NS5A or NS5B sequence data were discarded. In a final step, we retained only sequences from peer-reviewed publications with known sampling time and location. 

In addition, to reduce the size of the dataset without losing accuracy when estimating the quantity of between-country migration flows, within-country migration networks were represented by a randomly chosen taxon of that network. The latter were identified as clades with at least 95% bootstrap support for which all taxa shared their sampling location. To this end, maximum likelihood trees were inferred from 1,000 bootstrapped alignments with RAxML v.8.2 under a GTR + Γ substitution model. All well-supported clades consisting exclusively of sequences from the same country were reduced to a single randomly selected strain from these clades, except for Spanish clades. For the NS3, NS5A and NS5B datasets, respectively 391, 163 and 22 sequences were removed. The resulting alignments covered 513 nt of NS3, 258 nt of NS5A and 312 nt of NS5B and contained 1,554, 1,373 and 1,071 sequences, respectively. The NS5A and NS5B alignments were shorter than the NS3 alignment because the amino acids relevant for drug resistance in NS5A are located only within domain I of the protein and because of the short length of the commonly sequenced conserved region within NS5B, targeted to determine the correct HCV genotype. A detailed overview of the datasets is provided in Table 1.

**Table 1:** Composition of hepatitis C virus subtype 1a gene-specific datasets, 1989–2016 (n = 3,998)

Region	Country	NS3		NS5A		NS5B	
		n	%	n	%	n	%
Anglosphere	Australia	37	2.4	19	1.4	40	3.7
	India	NA		NA		16	1.5
	New Zealand	49	3.2	NA		49	4.6
	Pakistan	1	0.1	1	0.1	1	0.1
	**Regional total **	**87**	**5.6**	**20**	**1.5**	**106**	**9.9**
Europe (excluding Spain)	Belgium	18	1.2	NA		3	0.3
	Cyprus	NA		NA		14	1.3
	France	437	28.1	5	0.4	5	0.5
	Germany	105	6.8	9	0.7	23	2.1
	Italy	129	8.3	68	5.0	41	3.8
	Netherlands	1	0.1	1	0.1	81	7.6
	Sweden	NA		38	2.8	NA	
	Switzerland	44	2.8	45	3.3	45	4.2
	United Kingdom	4	0.3	NA		117	10.9
	**Regional total**	**738**	**47.5**	**166**	**12.1**	**329**	**30.7**
Far East	China	1	0.1	1	0.1	31	2.9
	Japan	1	0.1	NA		NA	
	Thailand	17	1.1	17	1.2	NA	
	Vietnam	NA		NA		18	1.7
	**Regional total**	**19**	**1.2**	**18**	**1.3**	**49**	**4.6**
Middle East	Iran	NA		NA		8	0.7
	Saudi Arabia	NA		NA		41	3.8
	**Regional total**	**NA**		**NA**		**49**	**4.6**
North America	Canada	2	0.1	3	0.2	3	0.3
	United States	370	23.8	1,037	75.5	472	44.1
	**Regional total**	**372**	**23.9**	**1,040**	**75.7**	**475**	**44.4**
South America	Brazil	101	6.5	46	3.4	NA	
	Uruguay	NA		1	0.1	1	0.1
	**Regional total**	**101**	**6.5**	**47**	**3.4**	**1**	**0.1**
Spain	Spain	237	15.3	82	6.0	62	5.8
**Total**		**1,554**	100	**1,373**	100	**1,071**	100

NA: not available.

Sequence data from 24 countries contributed to at least one gene dataset. All data from Spain were newly generated in the context of drug resistance testing between 2014 and 2016. Countries were grouped into seven regions to minimise the impact of potential sampling biases. Listed are the absolute number of sequences per country (n) and relative proportion (%) with respect to the total number of sequences in the dataset. Cells marked NA indicate that no sequence data from this country was available to include for a particular dataset.

### Phylogenetic and phylogeographic inference

To efficiently reconstruct the history of viral spread from large datasets, we created a pipeline in which fast maximum likelihood-based tree estimation, rooting and branch length rescaling methods were combined with a flexible and scalable Bayesian discrete phylogeographic approach. Briefly, uncertainty about the reconstructions was taken into account by conditioning the phylogeographic reconstructions on the time-calibrated phylogenies estimated from 1,000 bootstrapped alignments. For more details on the pipeline, we refer to the Supplementary material.

The timed trees were used to infer the migration processes with a model that allows for different migration rates to and from locations (i.e. the migration rate from location A to location B is allowed to be different from the migration rate in the opposite direction). The relative importance of the sampling locations varies across the gene-specific datasets (Table 1), which will affect the estimated intensities of virus movements between locations [16,17]. Following Faria et al. [18], we attempted to mitigate the impact of sampling heterogeneity by combining the geographical information from the three gene datasets when reconstructing the history of virus spread. As it is well established that the origin of all currently circulating HCV1a lineages is on the North American continent [4,19], we used the posterior probability (PP; this can be interpreted as the probability that the reconstructed location is true given the data and the model) of North America as root state location to evaluate whether simultaneously drawing information from multiple datasets improved the accuracy of the spatiotemporal estimates. The impact of sampling heterogeneity was also minimised by grouping all taxa at a higher-order level. Our choice of grouping reflected the pragmatic need to mitigate the impact of the sampling process on the inferred migration links without resorting to an overly coarse categorisation. The grouping was also informed by the literature and was designed to capture relevant higher-level migration flows. Specifically, we took into account (i) that the origins of the current HCV1a diversity lie in North America [4,19], (ii) that there are cultural and/or historical links – and therefore indirectly perhaps also pathogen migration links [20] – between Spain and other countries in Europe as well as between Spain and Latin America [21], and (iii) that HCV1a lineages are mainly transmitted by PWID [22] and it is therefore possible that routes of virus spread might overlap with international drug trafficking routes [23]. We chose to group the samples in the following categories (Table 1): Spain, Europe excluding Spain (all non-Spanish isolates from Europe), North America (accounting for the United States and Canada), Anglosphere (all English-speaking countries except for those in North America and the United Kingdom), Far East, Middle East and South America. 

A model-averaging procedure (the Bayesian stochastic search variable selection procedure) was used to identify the migration pathways that are most relevant in the history of spread, while SpreaD3 [24] was used to calculate the statistical support (expressed as Bayes factors (BF)) for all possible types of virus movements. Estimates of the number of migration events between all sampled locations were obtained by probabilistically mapping the evolution of location state changes onto the phylogenies [25]. These estimates were post-processed using scripts developed in house in the Perl and R programming languages to derive the proportions of specific types of migration events and their dynamics through time.

The size structure of transmission networks in the HCV1a epidemic in Spain was evaluated with a phylogeographic rarefaction curve. This is a plot of the expected number of introduction events into a specific location as a function of the number of taxa randomly selected from all taxa sampled at that location [3]. The plot was created from 250 spatially annotated phylogenies. Because each introduction event marks the start of a local transmission network, and the probability of detecting a previously unrecognised introduction event hence depends both on the number of Spanish transmission networks in the sample and on their relative abundance, the gradient of the slope holds epidemiologically relevant information. In one extreme, all sampled viruses from Spain originate from a single transmission network and the curve will have a zero-degree slope as additional isolates represent the already detected network. In the other extreme, when all isolates represent different transmission networks, the curve is expected to have a constant slope.

### Ethical statement

The Ethics Committee of the San Cecilio Hospital, Granada, approved the study. No informed consent was required as patient information was anonymised and de-identified before analysis.

## Results

### Three samples of the hepatitis C virus subtype 1a evolutionary history

The varying geographic composition of the NS3, NS5A and NS5B datasets (Table 1) implied that they may capture varying details of the epidemic spread of HCV1a in more detail than the other datasets do. For example, the contribution of European and North American data to the NS5B dataset was smaller (69.5%) than their contribution to the NS3 (86.5%) and NS5A (93.6%) datasets, probably a consequence of the intensive global use of this gene region for subtyping purposes. Therefore, NS5B may provide a more accurate view of migration patterns involving non-Western countries. In contrast, the NS3 and NS5A datasets contained sequence information for countries for which no NS5B data were available.

**Figure 1:**
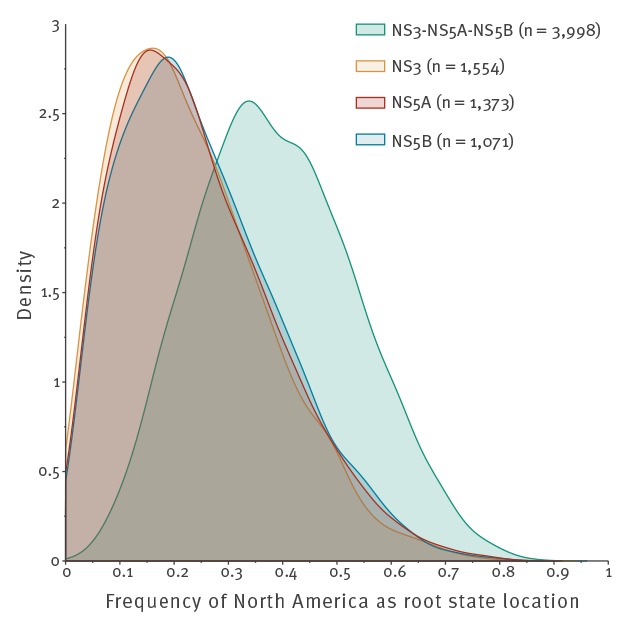
Support for North America as the origin of all currently circulating hepatitis C virus subtype 1a strains, 1989–2016 (n = 3,998)

When evaluating the impact of simultaneously drawing geographical information from the three gene datasets, support for North America as the root state location was notably higher, while not overwhelming, when compared with the estimates from the individual gene datasets (Figure 1). For this reason, we report here only results of analyses in which the location information is shared across the gene partitions.

The frequency distribution of locations at the root of the phylogenetic tree, or how likely the virus is to reside at a particular location at the start of the epidemic, is a parameter of the phylogeographic model, and the x-axis denotes this frequency for North America. The y-axis values or density values correspond to the proportion of trees for which a particular frequency was observed and can be thought of as the heights of the bars in a histogram. The shift of the density profile towards higher frequencies shows that the posterior probability of North America as the location of origin increases when sharing the geographical information across the gene datasets.

### North America and Europe as origins of hepatitis C virus subtype 1a in Spain

**Figure 2:**
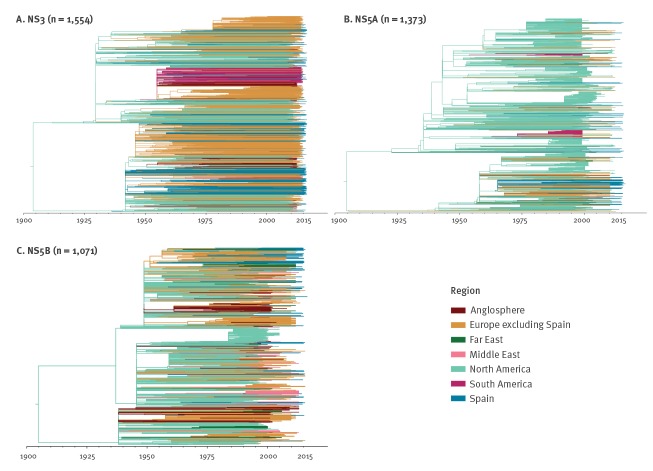
Global migration history of hepatitis C virus subtype 1a, estimated from the NS3, NS5A and NS5B gene fragments, ca 1904–2016 (n = 3,998)

The maximum clade credibility summary trees estimated from the three gene datasets were in line with North America being the origin of the current HCV1a pandemic. The many North American lineages that survived into the 21st century also show that North America continued to be an important source location throughout the epidemic history (Figure 2). Focusing on the locations for which the epidemiological linkage is well supported (Table 2), we find that North America was the dominant origin location of migration events (72.9%). Of these events, 12.5% were directed towards Spain. Europe was the second largest source, and 60.4% of the migrations from within the European continent were directed towards Spain. We also found good support (BF ≥ 50) for Spain as a source location for HCV1a in other European countries, but not for North America (Table 2). The imbalance in the number of incoming and outgoing virus movements (mean: 202; 95% highest posterior density (HPD): 142–258) shows that Spain acted as a sink for HCV1a in Europe. With the exception of a weakly supported link from Spain to South America (BF support of 4.7), no other pathways that involve Spain were supported by the data.

The branches in the time-scaled HCV1a maximum clade credibility trees constructed for the three genes are coloured by their most probable location state. The trunk lineages, marked in lighter green, reside in North America.

**Table 2:** Posterior probabilities for all types of hepatitis C virus subtype 1a migrations between the regional groupings, ca 1904–2016 (n = 3,998)

	To	Anglosphere	Europe excluding Spain	Far East	Middle East	North America	South America	Spain
From								
Anglosphere			**2.20**	0.48	0.04	0.51	0.17	0.08
Europe excluding Spain		**2.93**		**1.16**	0.11	**3.74**	0.44	**11.94**
Far East		0.12	0.07		0.02	0.10	0.02	0.02
Middle East		0.01	0.03	0		0.01	0.01	0.02
North America		**8.69**	**39.47**	**4.57**	**3.18**		0.66	**11.60**
South America		**1.26**	**1.56**	0.02	0	**1.29**		0.15
Spain		0.02	**3.11**	0.02	0.02	0.11	0.04	

Posterior probabilities (%) for migration rates supported by a Bayes factor ≥ 50 are marked in bold.

**Figure 3:**
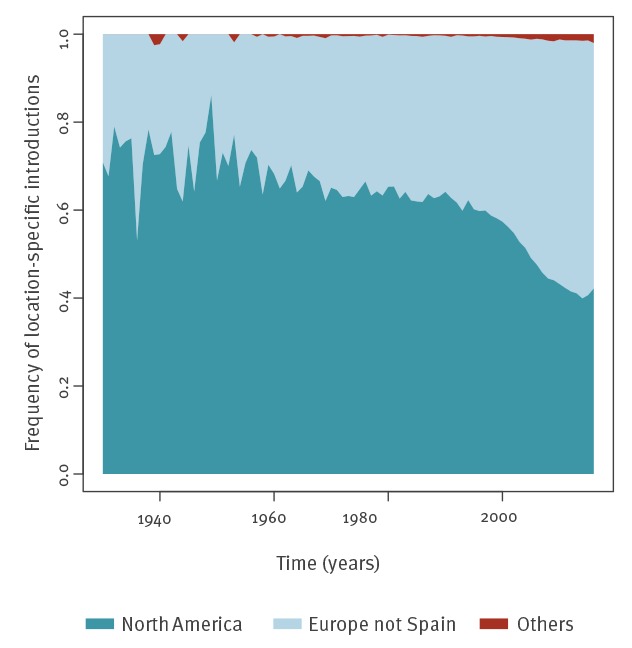
The importance of origin locations for hepatitis C virus subtype 1a varies during the twentieth century, Spain (n = 3,998)

To investigate temporal trends in the relative importance of North America and Europe as source locations for HCV1a migration to Spain, the frequency of introductions into Spain separated by origin location was plotted over time (Figure 3). This revealed a stable pattern throughout the largest part of the 20th century. However, near the end of the century, the frequency of introductions from overseas decreased in favour of other European countries (Figure 3). Figure 3 also visualises that the HCV1a epidemics in other regions in the world were contributed little (1.1%) to the Spanish one.

The plot shows the contribution of North America, Europe excluding Spain and ‘Others’ to the total number of HCV1a lineages imported to Spain. The x-axis denotes the time of HCV1a introductions to Spain. The continuing decline in the proportion of introductions from North America to Spain has been more pronounced in recent years.

### Numerous hepatitis C virus subtype 1a introductions fuel the Spanish epidemic

**Figure 4:**
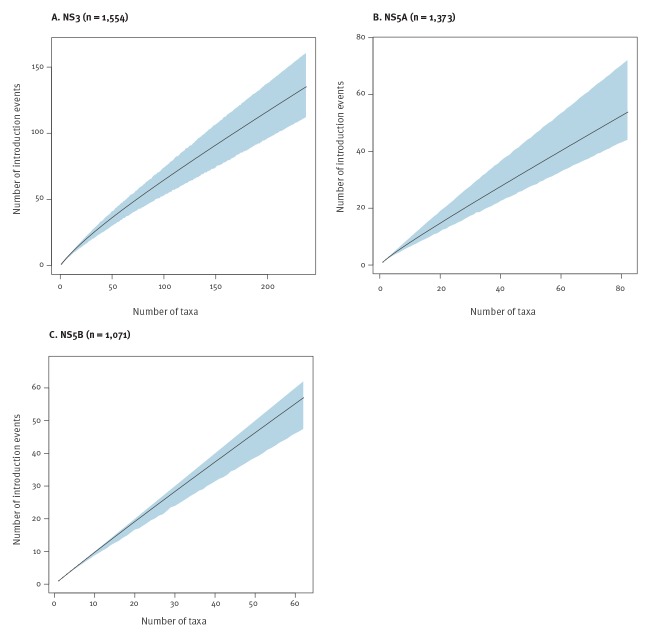
Phylogeographic rarefaction curves of the hepatitis C virus subtype 1a epidemic for the NS3, NS5A and NS5B gene datasets, Spain (n = 3,998)

We estimated a phylogeographic rarefaction curve for each of the gene segments to evaluate the size structure of the transmission network in more detail (Figure 4). For the three datasets, a similar pattern with an almost constant slope was observed. Our sample of the Spanish HCV1a epidemic thus indicates that there were many smaller, similar-sized transmission clusters rather than one or a few larger, dominant transmission networks. The absence of a clear inflection point shows that a larger sample will probably identify more introduction events. This pattern agreed with the absence of well-supported Spanish clusters in the NS5A dataset, and with a limited number in the NS5B (one 2-taxon cluster) and NS3 (five 2-taxon clusters) datasets (Supplementary Table S1).

The solid line represents the expected number of introduction events (y-axis) in Spain for n randomly chosen Spanish taxa (x-axis). The grey-shaded area marks the 95% Bayesian credible interval.

## Discussion

In this study, we have described the changing transmission flows that connect the Spanish HCV1a transmission networks with those elsewhere. Our results highlight that the Spanish HCV1a epidemic is of a diffuse nature because it is not structured as a small number of large transmission networks that were particularly successful in fuelling the spread of HCV1a in Spain. Instead, the circulation patterns were shaped by many introduction events from abroad, particularly from other European countries and North America. Hence, the outcome of Spanish efforts to control HCV1a spread is also dependent on reducing the prevalence in the groups that are at most risk in other European countries and North America. Given that PWID are the major source of ongoing HCV transmission in these countries [26], this calls for an intensification of close follow-up and treatment in this population, as well as needle exchange and opioid substitution programmes. Of note, the absence of clear phylogenetic structuring of other HCV subtypes in European PWID by geographical region indicates that the pattern that we recovered for subtype 1a may be a general one [27].

The link between pathogen dispersal routes and timing, and the intensity of human migration fluxes is well established [20]. Traffic is less intense between South America and Europe (including Spain) than between North America and Europe (including Spain). That the spread of HCV1a in South America [28] is more recent than in North America, the cradle of all current HCV1a lineages [4,19], probably accounts for the absence of substantial import of HCV1a into Spain from South America (BF < 50). The changing importance of North America and Europe as dominant source locations for new HCV1a lineages also links to shifts in the destination of historic migration waves. At the beginning of the 20th century, there was a large outflow from Spain to, and re-migration from, North America, whereas from the 1950s onwards, emigration was predominantly directed to European destinations [21,29]. Temporal changes in the intensity of other activities that involve cross-border interactions with other European countries such as trade and tourism are also in line with this trend [30].

During the second half of the 20th century, there was a continuous improvement in medical procedures, paralleled by other societal changes that resulted in a shift from mostly iatrogenic spread to an epidemic with injecting drug use as the main risk factor for HCV1a transmission in resource rich settings [26], including Spain [12]. Unfortunately, we could not directly assess this because data on risk behaviour were not available. However, most patients (69%) from Spain were sampled in 2014 and 2015, when drug resistance testing was predominantly available to infectious disease specialists; these patients were therefore mainly HIV/HCV-co-infected. From late 2015 onwards, requests for drug resistance testing shifted towards hepatologists, which implies that from then on, most tested patients were HIV-negative. As injecting drug use is by far the predominant risk behaviour (> 75%) for HIV/HCV-co-infected patients in Spain and 70% of the HIV-negative HCV1a patients are infected through injecting drug use ([12] and data not shown), our dataset most likely to a large extent captures the HCV1a dynamics among PWID. Knowledge of the age distribution of the Spanish patients would allow us to pinpoint more precisely to what extent drug user networks and re-migration of former economic migrants [31] are responsible for the increased influx of HCV1a from within Europe. This type of information is commonly available for evolutionary investigations of the HIV epidemiology [32]. The ability of such information to assist in identifying sub-populations at increased risk of HCV infection or sub-populations that disproportionally contribute to HCV spread will undoubtedly stimulate its use in future studies [33]. Note that the small number of clusters that represent HCV transmission within Spain a priori prevent a confident assessment of the circulation within the country but not between countries, and of the infection dynamics within different demographic and behavioural subgroups. Hence, the observation that in five of six pairs, both samples originated from the south of Spain should be interpreted with caution.

Confidence in the inferred evolutionary relationships generally increases with increasing sequence lengths. It is thus of interest to sequence longer stretches of the virus genome. Although the precision of estimates can also be boosted by concatenating multiple genomic fragments [34], we chose not to do so here because for most patients, only data from one gene segment was available. Pinpointing the geographic origin of imported lineages more precisely is useful when testing epidemiological hypotheses, but we did not pursue this here because the contribution of many European countries to the gene datasets was limited and variable. In fact, we grouped the sampling locations by region specifically to counteract the influence of geographical imbalances in the datasets. This, and sharing the geographical information across the datasets, improved the ancestral reconstructions. Nonetheless, the frequency with which North America was inferred as root state location (mean: 38%; 95% HPD: 10.3–66.2) was not high. We believe that the short length of the alignments and the consequently high phylogenetic uncertainty, combined with stochastic error in the estimation of the locations, adequately explain this level of uncertainty, although a bias may remain despite all precautions. The structured coalescent represents an attractive alternative to this problem, but the computational demands imposed by the size and complexity of datasets such as those used in this study are currently still prohibitive [16]. Alternatively, a possible confounding effect of sampling biases can also be prevented by incorporating external epidemiological data [35].

In summary, we found that the HCV1a epidemic in Spain was heavily mixed with epidemics elsewhere. This provides a contemporaneous rationale for concerted anti-HCV actions in Spain, Europe and, to a somewhat lesser extent, North America.

## Supplementary Data

490000 Supplement
