# Supplementary material for: Increasing importance of European lineages in seeding the hepatitis C virus subtype 1a epidemic in Spain
Source: Euro Surveill. 2019 Feb 28;24(9):1800227. doi: 10.2807/1560-7917.ES.2019.24.9.1800227 (PMC6402173; doi:10.2807/1560-7917.ES.2019.24.9.1800227)
Supplement: Supplement [file 1800227_CUYPERS_Supplement.pdf]

Supplement

"This supplementary material is hosted by *Eurosurveillance* as supporting information alongside the article [Increasing importance of European lineages in seeding the hepatitis C virus subtype 1a epidemic in Spain] on behalf of the authors who remain responsible for the accuracy and appropriateness of the content. The same standards for ethics, copyright, attributions and permissions as for the article apply. *Eurosurveillance* is not responsible for the maintenance of any links or email addresses provided therein."

Methods

Newly generated sequence data for the hepatitis C virus from Spain

Briefly, the clinical samples from Spanish patients were subjected to RNA extraction in a Magnapure Compact system (Roche Diagnostics) according to the manual, followed by a randomly primed cDNA synthesis (ThermoScientific). cDNA was used for independent amplification of NS3, NS5A and NS5B, with the primers and PCR conditions provided in the table below. DNA sequencing was performed using the same primers as for the PCR amplification using an ABI Prism 3500 analyzer.

Table 1. PCR primers and conditions for the amplification of the NS3, NS5A and NS5B genes for the HCV subtype 1a.

Gene	PRIMERS	PCR Profile		
NS3	**Forward 5'** GGAATGGTCTCCAAGGGGTGGA **3'**<br>**Reverse 5'** CATGGGCCTTGGACATGTAAGC **3'**	94<br>*94*<br>*58*<br>*72*<br>72	3'<br>*1'*<br>*1'*<br>*70''*<br>5'	 *x43*
NS5A	**Forward 5'** TCCTGGYTRAGGGACATCT **3'**<br>**Reverse 5'** CCGTGGTGTAGGCRTTRAT **3'**	94<br>*94*	3'<br>*30''*	 *x35*

		60<br>*72*<br>72	*30''*<br>*45''*<br>5'	
NS5B	**Forward 5'** CNTAYGAYACCMGNTGYTTTGACTC **3'**<br>**Reverse 5'** TTNGADGAGCADGATGTWATBAGCTC **3'**	95<br>*94*<br>*56*<br>*72*<br>72	2'<br>*20''*<br>*30''*<br>*1'*<br>10'	 *x45*

Phylogenetic pipeline

To efficiently reconstruct the virus's migration history from large datasets, we created a software pipeline in which fast maximum likelihood-based tree estimation, rooting and branch length rescaling methods are tied to a flexible and scalable Bayesian discrete phylogeographic approach. The pipeline starts with estimating maximum likelihood phylogenies from 1000 bootstrapped alignments using RAxML v.1.8.2, allowing for among-site-variation under the GTR nucleotide substitution model (*1*). The resulting undirectional trees are then rooted under the assumption of a strict molecular clock with the rtt (root-to-tip) function in Ape version 3.4 in R v3.1.3 (R core Team 2012). Next, the tips are pinned to their sampling date with treedater (*2*). For this we again assumed a constant rate of evolution, which was constrained to the lower and upper limits of the 95% Bayesian Credible Interval (BCI) interval of the gene-specific HCV subtype 1a evolutionary rate estimates derived from a dataset with strong time signal (*3*). Specifically, the substitution rate (expressed as number of nucleotide substitutions (x10^{-3}) per site per year) was constrained between 0.73 and 1.28 for NS3, between 0.97 and 1.73 for NS5A and between 0.76 and 1.35 for NS5B. This, however, results in timings of the HCV1a most recent common ancestor (for all three datasets) that are incompatible with the known origins. To compensate for the lack of clear temporal structure of the datasets, we explicitly incorporated information on the age of the HCV1a epidemic, which was estimated around 1908 (95% BCI: 1897-1919) using BEAST v1.8.4 (*4*). Dataset and

evolutionary model details for this analysis are available from the authors upon request. Using this information, the branch lengths were rescaled such that the root node age corresponds to an epidemic age drawn at random from a normal distribution centered at 1908 and with a standard deviation such that the 2.5 and 97.5 percentiles correspond to 1897 and 1919.

The Bayesian Evolutionary Analysis by Sampling Trees (BEAST) software was used for all phylogeographic analyses (v.1.8.4, *4*) and BEAGLE (*5*) served to improve the computational performance. Using this framework for Bayesian phylogenetics, a sample of plausible reconstructions of the phylogeographic history is obtained through Markov chain Monte Carlo (MCMC) sampling (see also *6*). Three Markov chains were run for 50 million states and samples were logged every 10.000 states. Convergence and mixing properties were assessed with Tracer v.1.7 (*7*), and the post burn-in samples were combined and jointly analysed. Maximum clade credibility (MCC) trees were summarized with TreeAnnotator v1.8.4 and visualized with FigTree v. 1.4.3 (http://tree.bio.ed.ac.uk/software/figtree/).

References

1. Stamatakis A. RAxML version 8: a tool for phylogenetic analysis and post-analysis of large phylogenies. Bioinformatics. 2014;30:1312-3.
2. Volz EM, Frost SDW. Scalable relaxed clock phylogenetic dating. Virus Evolution. 2017;3.
3. Al-Qahtani AA, Baele G, Khalaf N, Suchard MA, Al-Anazi MR, Abdo AA, *et al*. The epidemic dynamics of hepatitis C virus subtypes 4a and 4d in Saudi Arabia. Sci Rep. 2017;7:44947.

4. Drummond AJ, Suchard MA, Xie D, Rambaut A. Bayesian phylogenetics with BEAUti and the BEAST 1.7. Mol Biol Evol. 2012;29:1969-73.
5. Suchard MA, Rambaut A. Many-core algorithms for statistical phylogenies. Bioinformatics. 2009;25:1370-6.
6. Nascimento FF, Reis MD, Yang Z. A biologist's guide to Bayesian phylogenetic analysis. Nat Ecol Evol. 2017;1:1446-54.
7. Rambaut A, Drummond AJ, Xie D, Baele G, Suchard MA. Posterior summarization in Bayesian phylogenetics using Tracer 1.7. Syst Biol. 2018;67:901-904.

FIGURE S1. The newly generated sequences originate from patients attending a variety of hospitals across Spain, Spain, 2014-2016 (n = 272).

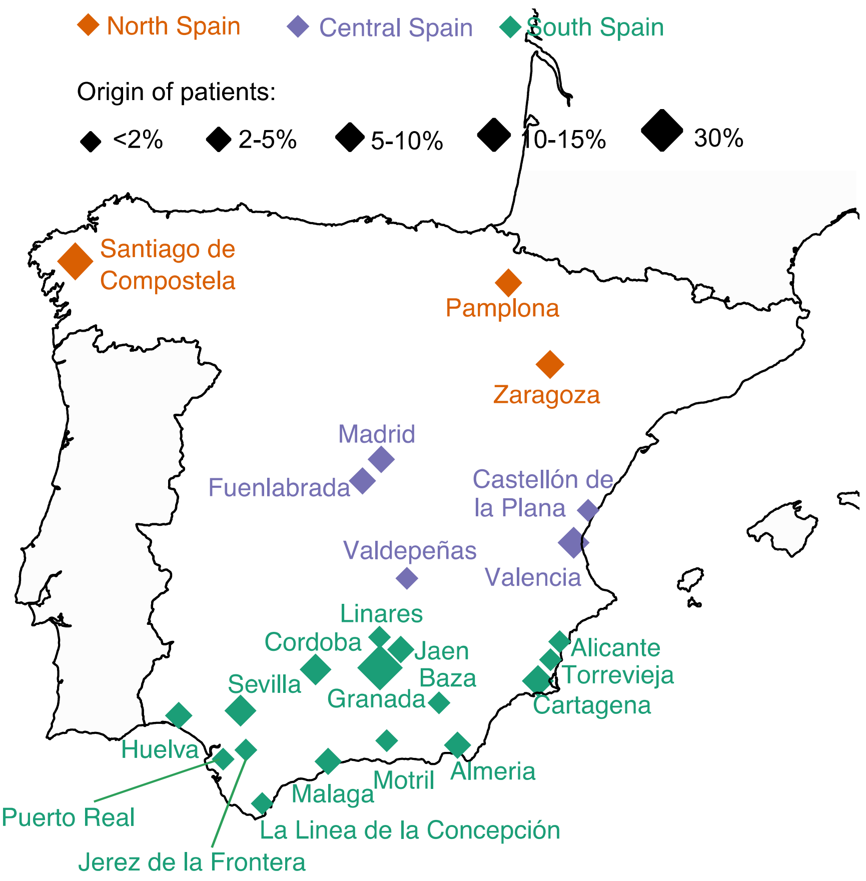

Viral genetic data was obtained in 24 different cities across Spain, classified as North Spain (orange), Central Spain (purple) and South Spain (green). Regional information was available for most patients (272/283). The proportion of patients originating from a location is visualized on the map, represented by the size of the diamond, as indicated in the legend.

TABLE S1. The Spanish clusters in the NS3 and NS5B datasets with root node posterior probability ≥99%, Spain, 2014 – 2016 (n = 283).

Gene	Cluster	Location of taxon 1	Location of taxon 2	Cluster support
NS3	1	Granada (South)	Granada (South)	100
	2	Jerez de la Frontera (South)	Jerez de la Frontera (South)	100
	3	Valencia (Central)	Spain (city or region not known)	100
	4	Cartagena (South)	Cartagena (South)	100
	5	Huelva (South)	Huelva (South)	99
NS5B	1	Cartagena (South)	Cartagena (South)	99

Well-supported clusters were not identified in the NS5A dataset. The only available metadata information for the taxa involved was their sampling location.

TABLE S2. List of accession numbers of all publicly available NS3, NS5A and NS5B viral genetic sequences used in this study, global dataset, 1989 – 2016 (n = 3,617).

**NS3** (n = 1,317)		**NS5A** (n = 1,291)		**NS5B** (n = 1,009)	
KX621422	JN704295	KX621544	JQ062155	KX621422	JX463638
KX621427	JN704294	KX621543	JQ062157	KX621423	JX463637
KX621430	JN704293	KX621542	JQ062160	KX621424	JX463635
KX621435	JN704291	KX621540	JQ062207	KX621427	JX463634
KX621436	JN704290	KX621538	JQ062208	KX621430	JX463633

KX621438	JN704286	KX621536	JQ062209	KX621435	JX463632
KX621439	JN704285	KX621535	JQ062210	KX621436	JX463631
KX621440	JN704284	KX621533	JQ062211	KX621437	JX463630
KX621442	JN704283	KX621532	JQ062212	KX621438	JX463629
KX621443	JN704282	KX621529	JQ062215	KX621439	JX463628
KX621444	JN704281	KX621528	JQ062216	KX621440	JX463627
KX621445	JN704280	KX621526	JQ062217	KX621442	JX463626
KX621448	JN704278	KX621525	JQ062221	KX621443	JX463625
KX621449	JN704277	KX621524	JQ062223	KX621444	JX463624
KX621450	JN704276	KX621523	JQ062224	KX621445	JX463622
KX621451	JN704275	KX621521	JQ062225	KX621448	JX463621
KX621455	JN704274	KX621517	JQ062273	KX621449	JX463620
KX621456	JN704273	KX621515	JQ062278	KX621450	JX463619
KX621457	JN704269	KX621514	JQ062315	KX621451	JX463618
KX621458	JN704268	KX621511	JQ062320	KX621452	JX463617
KX621460	JN704267	KX621510	JQ062321	KX621455	JX463616
KX621461	JN704266	KX621509	JQ062322	KX621456	JX463615
KX621462	JN704264	KX621505	JQ062323	KX621457	JX463614
KX621463	JN704263	KX621504	JQ062324	KX621458	JX463613
KX621464	JN704262	KX621500	JQ062365	KX621460	JX463611
KX621465	JN704261	KX621495	JQ062368	KX621461	JX463610
KX621467	JN704260	KX621485	KF667788	KX621462	JX463609
KX621468	JN704257	KX621484	KF667789	KX621463	JX463608
KX621471	JN704256	KX621482	KF667790	KX621464	JX463607
KX621473	JN704254	KX621474	KF667791	KX621465	JX463606
KX621474	JN704253	KX621473	KF667792	KX621466	JX463605
KX621482	JN704252	KX621471	KF667793	KX621467	JX463604
KX621484	JN704251	KX621468	KF667794	KX621468	JX463603
KX621495	JN704250	KX621467	KF667795	KX621471	JX463602
KX621500	JN704249	KX621466	KF667796	KX621473	JX463600
KX621504	JN704248	KX621465	KF667797	KX621474	JX463599
KX621505	JN704247	KX621464	KF667798	KX621482	JX463598
KX621509	JN704246	KX621463	KF667799	KX621484	JX463597
KX621510	JN704244	KX621462	KF667800	KX621485	JX463596
KX621511	JN704242	KX621461	KF667801	KX621495	JX463595
KX621512	JN704241	KX621460	KF667802	KX621500	JX463594
KX621514	JN704240	KX621459	KF667803	KX621504	JX463592
KX621515	JN704239	KX621457	KF667804	KX621505	JX463591
KX621517	JN704238	KX621456	KF667805	KX621509	JX463589
KX621521	JN704237	KX621455	KF667806	KX621510	JX463588
KX621523	JN704236	KX621452	KF667807	KX621511	JX463587
KX621524	JN704235	KX621451	KF667808	KX621512	JX463586
KX621525	JN704234	KX621450	KF667809	KX621514	JX463583
KX621526	JN704232	KX621449	KF667810	KX621515	JX463582
KX621528	JN704231	KX621448	KF667811	KX621517	JX463581
KX621529	JN704230	KX621446	KF667812	KX621520	JX463580
KX621532	JN704229	KX621444	KF667813	KX621521	JX463577
KX621533	JN704224	KX621443	KF667814	KX621523	JX463575
KX621536	JN704220	KX621442	KF667815	KX621524	JX463574
KX621538	JN704219	KX621440	KF667816	KX621525	JX463573
KX621540	JN704218	KX621439	KF667817	KX621526	JX463572
KX621542	JN704210	KX621438	KF667818	KX621528	JX463569
KX621543	JN704206	KX621437	KC283194	KX621529	JX463568
KX621544	JN704204	KX621436	KC844049	KX621532	JX463565
KP411688	JN704203	KX621435	JX463526	KX621533	JX463564
KP411687	JN704202	KX621430	JX463528	KX621535	JX463562
KP411665	JN704199	KX621427	JX463529	KX621536	JX463561
KP411617	JN704198	KX621424	JX463531	KX621538	JX463560
KP163204	JN704194	KX621423	JX463532	KX621542	JX463559
KP163178	JN704193	KX621422	JX463533	KX621543	JX463558

KP163151	JX472009	AY956465	JX463534	KX621544	JX463557
KP163171	JX472008	AY956466	JX463535	KR071886	JX463556
KP163165	JX472007	AY956468	JX463536	KR071931	JX463555
KP163020	JX472006	AY956469	JX463540	KR071898	JX463554
KP163046	JX472005	EU309586	JX463541	KR071904	JX463553
KP163233	KM580626	EU309589	JX463542	KR071936	JX463552
KP163230	KM580625	EU309593	JX463544	KC285245	JX463551
KP163484	KM580624	EU309594	JX463545	KU871306	JX463550
KP411698	KM580623	EU309597	JX463546	KU871305	JX463549
KP411716	KM580622	EU309600	JX463547	KU871304	JX463546
KP163499	KM580621	EU309609	JX463548	KU871303	JX463545
KP411719	KM580620	EU309615	JX463549	KU871302	JX463544
KP163442	KM580618	EU309616	JX463550	KU871301	JX463543
KP163239	KM580617	EU309617	JX463553	KU871300	JX463542
KP163214	KM580616	EU309618	JX463554	KT734522	JX463541
KP163051	KM580615	EU309619	JX463555	KJ437300	JX463539
KP163118	KM580614	EU309622	JX463556	KR072006	JX463538
KP163050	KM580613	EU309623	JX463557	KR072005	JX463537
KP163117	KM580612	EU309625	JX463558	KR072004	JX463536
KP162622	KM580611	EU309627	JX463559	KR072003	JX463534
KP163022	KM580609	EU309628	JX463560	KR072002	JX463533
KP409207	KM580608	EU309629	JX463561	KR072001	JX463532
KP163143	EU255992	EU309630	JX463562	KR072000	JX463531
KP163089	EU255991	EU309631	JX463563	KR071999	JX463530
KP409211	EU255990	EU309632	JX463564	KR071998	JX463529
KP409210	EU255989	EU309634	JX463565	KR071997	JX463528
KP409205	EU255988	EU309635	JX463566	KR071996	JX463527
KP409204	EU255987	EU309636	JX463568	KR071995	JX463526
KJ668268	EU255986	EU309638	JX463569	KR071994	JX463525
KJ668267	EU255985	EU309639	JX463570	KR071993	KC285252
KJ668266	EU255984	EU309641	JX463571	KR071992	KC285251
KJ668265	EU255983	EU309644	JX463573	KR071991	KC285250
KJ668264	EU255982	EU309645	JX463574	KR071990	KC285248
KJ668262	EU255981	EU309647	JX463575	KR071989	KC285247
KJ668261	EU255979	EU309651	JX463576	KR071988	KC285246
KJ668260	EU255978	EU309652	JX463577	KR071987	KC285198
KJ668259	EU255977	EU309655	JX463578	KR071986	KC844049
KJ668258	EU255976	EU309657	JX463579	KR071985	KC283194
KJ668257	EU255975	EU309658	JX463580	KR071984	KF667787
KJ668256	EU255974	EU309659	JX463581	KR071983	KF667785
KJ668255	EU255973	EU309661	JX463582	KR071982	KF667784
KJ668254	EU255971	EU309662	JX463583	KR071981	KF667783
KJ668253	EU255970	EU309663	JX463584	KR071980	KF667782
KJ668252	EU255969	EU309665	JX463585	KR071979	KF667781
KJ668251	EU255968	EU309666	JX463586	KR071978	KF667780
KJ668250	EU255966	EU309668	JX463587	KR071977	KF667779
KJ668249	EU255965	EU309669	JX463588	KR071976	KF667778
KJ668248	EU255964	EU309670	JX463589	KR071975	KF667777
KJ668247	EU255963	EU309671	JX463591	KR071974	KF667776
KJ668246	EU256074	EU309673	JX463592	KR071973	KF667775
KJ668245	EU256073	FJ958369	JX463594	KR071972	KF667774
KJ668244	EU256072	FJ958370	JX463595	KR071971	KF667773
KJ668243	EU256071	FJ958371	JX463597	KR071970	KF667772
KJ668242	EU256070	FJ958373	JX463598	KR071969	KF667771
KJ668241	EU256069	FJ958374	JX463599	KR071968	KF667770
KJ668240	EU256058	FJ958376	JX463600	KR071967	KF667769
KJ668239	EU256057	FJ958378	JX463602	KR071966	KF667768
KJ668238	EU256053	FJ958379	JX463603	KR071965	KF667767
KJ668237	EU256052	FJ958380	JX463604	KR071964	KF667766
KJ668236	EU256051	FJ958381	JX463606	KR071960	KF667765

KJ668235	EU256050	FJ958382	JX463607	KR071959	KF667764
KJ668234	EU256049	FJ958383	JX463608	KR071958	KF667763
KJ668233	EU256048	FJ958384	JX463610	KR071957	KF667762
KJ668232	EU256046	FJ958385	JX463611	KR071956	KF667761
KU871306	EU256044	FJ958386	JX463613	KR071954	KF667760
KU871305	EU256043	FJ958387	JX463615	KR071953	KF667759
KU871304	EU256040	FJ958388	JX463617	KR071950	KF667758
KU871303	EU256039	FJ958389	JX463619	KR071949	KF667757
KU871302	EU256038	FJ958390	JX463620	KR071948	KF667756
KU871301	EU256037	FJ958391	JX463622	KR071947	JQ062389
KU871300	EU256036	FJ958392	JX463624	KR071946	JQ062388
KT734522	EU256035	FJ958393	JX463625	KR071945	JQ062387
KJ437300	EU256034	FJ958394	JX463626	KR071944	JQ062386
KT735187	EU256033	FJ958395	JX463627	KR071943	JQ062385
KT735181	EU256032	FJ958396	JX463628	KR071942	JQ062384
KT735179	EU256031	FJ958397	JX463629	KR071941	JQ062383
KT735178	EU256030	FJ958398	JX463630	KR071940	JQ062382
KM874789	EU256029	FJ958399	JX463631	KR071939	JQ062329
KM874787	EU256028	FJ958400	JX463633	KR071938	JQ062328
KM587614	EU256027	FJ958403	JX463635	KR071937	JQ062325
KP411746	EU256026	FJ958404	JX463637	KR071935	JQ062237
KP411745	EU256025	FJ958406	JX463638	KR071934	JQ062236
KP411744	EU256024	FJ958408	EU155294	KR071933	JQ062235
KP411742	EU256023	FJ958409	EU155345	KR071932	JQ062234
KP411741	EU256022	FJ958410	EU260395	KR071930	JQ062233
KP411740	EU256021	FJ958412	EU239713	KR071929	JQ062232
KP411739	EU256020	FJ958414	EU239715	KR071928	JQ062230
KP411738	EU256019	FJ958415	EU239716	KR071926	JQ062229
KP411737	EU256018	FJ958416	EU250017	KR071925	JQ062228
KP411735	EU256017	FJ958417	EU260396	KR071924	JQ062227
KP411734	EU256016	FJ958418	EU234063	KR071923	JQ062168
KP411732	EU256015	FJ958419	EU234064	KR071922	JQ062167
KP411731	EU256014	FJ958420	EU155213	KR071921	JQ062165
KP411725	EU256013	FJ958421	EU155214	KR071920	JQ062163
KP411724	EU256012	FJ958422	EU155215	KR071919	JQ062162
KP411722	EU256011	FJ958423	EU155216	KR071918	JQ062161
KP411720	EU256010	FJ958424	EU155233	KR071917	JQ062104
KP411718	EU256009	FJ958425	EU155236	KR071916	JQ062103
KP411717	EU256008	FJ958426	EU155237	KR071915	JQ062101
KP411715	EU256007	FJ958427	EU155238	KR071914	JQ062099
KP411714	EU256006	FJ958428	EU155239	KR071913	JQ062097
KP411713	EU256005	FJ958429	EU155240	KR071912	JQ062096
KP411712	EU256004	FJ958431	EU155242	KR071911	JQ062095
KP411710	EU256003	FJ958432	EU155243	KR071910	JQ061986
KP411709	EU256002	FJ958433	EU155244	KR071909	JQ061985
KP411708	EU255999	FJ958434	EU155245	KR071908	JQ061984
KP411707	EU255998	FJ958435	EU155246	KR071907	JQ061983
KP411704	EU255997	FJ958436	EU482831	KR071906	JQ061981
KP411703	EU255996	FJ958437	EU482832	KR071905	JQ061980
KP411702	EU255995	FJ958438	EU482834	KR071903	JQ061970
KP411701	EU255994	FJ958439	EU482835	KR071902	JQ061966
KP411700	EU255993	FJ958440	EU482836	KR071901	JQ061961
KP411699	EU155347	FJ958441	EU482837	KR071900	JQ061958
KP411697	EU256107	FJ958443	EU482838	KR071899	JQ061957
KP411696	EU256106	FJ958444	EU482840	KR071897	JQ061956
KP411695	EU256105	FJ958446	EU482841	KR071896	JQ061914
KP411694	EU256104	FJ958447	EU482842	KR071895	JQ061913
KP411693	EU256097	FJ958448	EU482843	KR071894	JQ061912
KP411692	EU256096	FJ958449	EU482844	KR071893	JQ061911
KP411691	EU256095	FJ958452	EU482845	KR071892	JQ061910

KP411690	EU256094	FJ958453	EU482846	KR071891	JQ061802
KP411689	EU256087	FJ958454	EU482847	KR071890	JQ061787
KP411686	EU256068	FJ958455	EU482848	KR071889	JQ061786
KP411685	EU256067	FJ958456	EU482850	KR071888	JQ061785
KP411684	EU256060	FJ958457	EU482852	KR071887	JQ061783
KP411683	EU255958	FJ958459	EU482853	KR071885	JQ061781
KP411682	EU255957	FJ958460	EU482854	KR071884	JQ061776
KP411681	EU255956	FJ958461	EU482855	KR071883	JQ061774
KP411680	EU255955	FJ958462	EU482856	KT735187	JQ061391
KP411679	EU255954	FJ958463	EU482857	KT735186	JQ061390
KP411678	EU255953	FJ958465	EU482858	KT735181	JQ061389
KP411677	EU255952	FJ958466	EU482861	KT735179	JQ061386
KP411676	EU255951	FJ958467	EU482862	KT735178	JQ061354
KP411675	EU255950	FJ958468	EU482863	KM874786	JQ061349
KP411674	EU255948	FJ958470	EU482864	KT735857	JQ061348
KP411673	EU255947	FJ958471	EU482865	KT735849	JQ061347
KP411672	EU255946	FJ958472	EU482866	KT735833	JQ062102
KP411671	EU255945	FJ958473	EU482867	KT735830	JQ061388
KP411670	EU255944	FJ958474	EU482868	KT735742	KC155254
KP411669	EU255943	FJ958476	EU482869	KT735722	JQ914274
KP411668	EU255942	FJ958477	EU482870	KT735719	JQ914272
KP411667	EU255940	FJ958479	EU482871	KT735711	JQ914271
KP411666	EU255939	FJ958480	EU482872	KT735710	JX961066
KP411664	EU255938	FJ958482	EU482873	KT735708	JX961049
KP411663	EU255937	FJ958484	EU482876	KT735706	JX961039
KP411662	EU255936	FJ958485	EU482878	KT735703	JX961032
KP411661	EU255935	FJ958486	EU482882	KT735701	JX961021
KP411660	EU255934	FJ958488	EU482884	KT735694	JX961012
KP411659	EU255933	FJ958489	EU155248	KT735693	JX961006
KP411658	EU255932	FJ958490	EU155249	KT735686	JX961000
KP411657	EU255931	FJ958491	EU155250	KT735679	JX437280
KP411656	EU255930	FJ958492	EU155251	KT735672	JX437279
KP411655	EU255929	FJ958499	EU155252	KT735671	JX437278
KP411654	EU255928	FJ958500	EU155266	KT735670	JX437277
KP411653	EU255927	FJ958501	EU155267	KM587614	JX437275
KP411652	EU660385	FJ958504	EU155268	JX472009	JX437274
KP411651	EU660384	FJ958505	EU155269	JX472008	JX437273
KP411650	EU660383	FJ958506	EU155270	JX472007	JX437272
KP411648	EU595699	FJ958507	EU155271	JX472006	JX437271
KP411646	EU595698	FJ958508	EU155272	JX472005	JX437270
KP411645	EU595697	FJ958510	EU155274	EU255992	JX437269
KP411644	EU569722	FJ958511	EU155275	EU255991	JX437268
KP411643	EU529681	FJ958512	EU155276	EU255990	JX437266
KP411642	EU529680	FJ958513	EU155277	EU255989	JX437265
KP411641	EU529679	FJ958514	EU155278	EU255988	JX437264
KP411640	EU529678	FJ958515	EU155282	EU255987	JX437263
KP411639	EU529677	FJ958516	EU155283	EU255986	JX437262
KP411638	EU529676	FJ958519	EU155284	EU255985	JX437261
KP411637	EU234065	FJ958521	EU155285	EU255984	JX437260
KP411636	EU155380	FJ958522	EU155286	EU255983	JX437259
KP411635	EU155378	FJ958524	EU155287	EU255982	JX437258
KP411634	EU155355	FJ958525	EU155288	EU255981	JX437257
KP411633	EU155354	FJ958526	EU155289	EU255980	JX437255
KP411632	EU155353	FJ958529	EU155290	EU255979	JX437254
KP411631	EU155352	FJ958532	EU155291	EU255978	JX437253
KP411630	EU155351	FJ958533	EU155292	EU255977	JX437252
KP411629	EU155349	FJ958535	EU155293	EU255976	JX437251
KP411628	EU155348	FJ958537	EU155295	EU255975	JX437249
KP411627	EU155346	FJ958538	EU155296	EU255974	JX437248
KP411626	EU155344	FJ958539	EU155297	EU255973	JX437246

KP411624	EU155343	FJ958540	EU155298	EU255971	JX437245
KP411623	EU155342	FJ958542	EU155299	EU255970	JX437244
KP411622	EU155341	FJ958543	EU155309	EU255969	JX437243
KP411621	EU155340	FJ958544	EU155310	EU255968	JX437242
KP411620	EU155339	FJ958545	EU155311	EU255966	JX437241
KP411619	EU155338	FJ958546	EU155313	EU255965	JX437237
KP411618	EU155323	FJ958547	EU155314	EU255964	JX437236
KP411616	EU155322	FJ958548	EU155319	EU255963	JX437233
KP411615	EU155321	FJ958549	EU155320	EU256074	JX437232
KP411614	EU155320	FJ958550	EU155321	EU256073	JX437231
KP163547	EU155319	FJ958551	EU155322	EU256072	JX437230
KP163540	EU155314	FJ958552	EU155323	EU256071	JX437229
KP163539	EU155313	FJ958554	EU155338	EU256070	JX437228
KP163536	EU155311	FJ958555	EU155339	EU256069	JX437227
KP163534	EU155310	FJ958556	EU155340	EU256058	JX437226
KP163533	EU155309	FJ958558	EU155341	EU256057	JX437225
KP163531	EU155299	FJ958559	EU155342	EU256053	JX437224
KP163529	EU155298	FJ958560	EU155343	EU256052	JX437223
KP163527	EU155297	FJ958561	EU155344	EU256051	JX437221
KP163525	EU155296	FJ958562	EU155346	EU256050	JX437220
KP163524	EU155295	FJ958563	EU155348	EU256049	JX437218
KP163523	EU155293	FJ958564	EU155349	EU256048	JX437217
KP163522	EU155292	FJ958566	EU155351	EU256047	JX437215
KP163517	EU155291	FJ958571	EU155352	EU256046	JX437214
KP163516	EU155290	FJ958573	EU155353	EU256044	JX437213
KP163513	EU155289	FJ958575	EU155354	EU256043	JX437212
KP163508	EU155288	FJ958578	EU155355	EU256041	JX437211
KP163507	EU155287	FJ958579	EU155378	EU256040	JX437208
KP163506	EU155286	FJ958580	EU155380	EU256039	JX437207
KP163505	EU155285	FJ958582	EU234065	EU256038	JX437206
KP163504	EU155284	FJ958583	EU529676	EU256037	JX437205
KP163503	EU155283	FJ958584	EU529677	EU256036	JX437204
KP163496	EU155282	FJ958585	EU529678	EU256035	JX437203
KP163494	EU155278	FJ958586	EU529679	EU256034	JX437202
KP163493	EU155277	FJ958587	EU529680	EU256033	JX437200
KP163486	EU155276	FJ958588	EU529681	EU256032	JX437199
KP163485	EU155275	FJ958589	EU569722	EU256031	JX437198
KP163482	EU155274	FJ958590	EU569723	EU256030	JX437197
KP163479	EU155272	FJ958591	EU595697	EU256029	JX437196
KP163477	EU155271	FJ958592	EU595698	EU256028	JX437195
KP163476	EU155270	FJ958593	EU595699	EU256027	JQ303593
KP163475	EU155269	FJ958594	EU660383	EU256026	EU684659
KP163470	EU155268	FJ958595	EU660384	EU256025	EU684657
KP163466	EU155267	FJ958596	EU660385	EU256024	EU684648
KP163463	EU155266	FJ958597	EU255927	EU256023	EU684632
KP163462	EU155252	FJ958599	EU255928	EU256022	EU684627
KP163453	EU155251	FJ958600	EU255929	EU256021	EU684618
KP163445	EU155250	FJ958602	EU255930	EU256020	EU684617
KP163439	EU155249	FJ958604	EU255931	EU256019	EU684600
KP163438	EU155248	FJ958605	EU255932	EU256018	EU684596
KP163436	EU482884	FJ958607	EU255933	EU256017	JF424754
KP163433	EU482882	FJ958608	EU255934	EU256016	JF424753
KP163432	EU482878	FJ958609	EU255935	EU256015	JF424752
KP163420	EU482876	FJ958611	EU255936	EU256014	JF424751
KP163419	EU482873	FJ958613	EU255937	EU256013	JF424750
KP163417	EU482872	FJ958615	EU255938	EU256012	JF424749
KP163416	EU482871	FJ958616	EU255939	EU256011	JF424748
KP163415	EU482870	FJ958617	EU255942	EU256010	JF424747
KP163413	EU482869	FJ958619	EU255943	EU256009	JF424746
KP163407	EU482868	FJ958620	EU255944	EU256008	JF424745

KP163406	EU482867	FJ958622	EU255945	EU256007	JF424744
KP163400	EU482866	FJ958623	EU255946	EU256006	JF424743
KP163399	EU482865	FJ958624	EU255947	EU256005	JF424742
KP163398	EU482864	FJ958625	EU255948	EU256004	JF424741
KP163395	EU482863	FJ958626	EU255949	EU256003	JF424740
KP163391	EU482862	FJ958627	EU255950	EU256002	JF424739
KP163389	EU482861	FJ958628	EU255951	EU255999	JF424738
KP163387	EU482858	FJ958629	EU255952	EU255998	JF424737
KP163386	EU482857	FJ958630	EU255953	EU255997	JF424736
KP163385	EU482856	FJ958631	EU255954	EU255996	JF424735
KP163383	EU482855	FJ958634	EU255955	EU255995	JF424734
KP163377	EU482854	FJ958641	EU255956	EU255994	JF424733
KP163376	EU482853	FJ958642	EU255957	EU255993	JF424732
KP163375	EU482852	FJ958644	EU255958	EU155347	JF424731
KP163374	EU482850	FJ958647	EU256060	EU256107	JF424730
KP163372	EU482848	FJ958653	EU256067	EU256106	JF424729
KP163371	EU482847	FJ958655	EU256068	EU256105	JF424728
KP163369	EU482846	FJ958657	EU256087	EU256104	JF424727
KP163367	EU482845	FJ958660	EU256094	EU256097	JF424726
KP163364	EU482844	FJ958670	EU256095	EU256096	JF424725
KP163363	EU482843	FJ958673	EU256096	EU256095	JF424724
KP163361	EU482842	FJ958675	EU256097	EU256094	JF424723
KP163360	EU482841	FJ958677	EU256104	EU256087	HQ537059
KP163355	EU482840	FJ958678	EU256105	EU256068	HQ537058
KP163353	EU482838	FJ958679	EU256106	EU256067	HQ537057
KP163352	EU482837	FJ958680	EU256107	EU256060	HQ537052
KP163351	EU482836	FJ958681	EU155347	EU255958	HQ537050
KP163350	EU482835	FJ958682	EU255993	EU255957	EU934906
KP163349	EU482834	FJ958683	EU255994	EU255956	FJ768905
KP163348	EU482832	FJ958685	EU255995	EU255955	FJ768831
KP163347	EU482831	FJ958688	EU255996	EU255954	FJ768829
KP163346	EU155246	FJ958690	EU255997	EU255953	FJ768828
KP163344	EU155245	FJ958691	EU255998	EU255952	FJ768827
KP163340	EU155244	FJ958693	EU255999	EU255951	FJ768826
KP163338	EU155243	FJ958694	EU256002	EU255950	FJ768825
KP163334	EU155242	FJ958696	EU256003	EU255949	FJ768824
KP163333	EU155240	FJ958698	EU256004	EU255948	FJ768823
KP163332	EU155239	FJ958700	EU256005	EU255947	FJ768822
KP163331	EU155238	FJ958702	EU256006	EU255946	FJ768821
KP163328	EU155237	FJ958703	EU256007	EU255945	FJ768820
KP163327	EU155236	FJ958705	EU256008	EU255944	FJ768819
KP163326	EU155233	FJ958708	EU256009	EU255943	FJ768818
KP163324	EU155216	FJ958710	EU256010	EU255942	FJ768817
KP163317	EU155215	FJ958715	EU256011	EU255941	FJ768816
KP163316	EU155214	FJ958716	EU256012	EU255940	FJ768815
KP163315	EU155213	FJ958718	EU256013	EU255939	FJ768814
KP163314	EU234064	FJ958719	EU256014	EU255938	EU081327
KP163312	EU234063	FJ958720	EU256015	EU255937	AY956469
KP163311	EU260396	FJ958725	EU256016	EU255936	AY956468
KP163306	EU250017	FJ958759	EU256017	EU255935	AY956466
KP163305	EU239716	FJ958760	EU256018	EU255934	AY956465
KP163302	EU239715	FJ958761	EU256019	EU255933	KU563482
KP163299	EU239713	FJ958762	EU256020	EU255932	KU563481
KP163298	EU260395	FJ958763	EU256021	EU255931	KU563480
KP163295	EU155345	FJ958764	EU256022	EU255930	KU563479
KP163294	EU155294	FJ958765	EU256023	EU255929	KU563478
KP163291	JX463641	FJ958767	EU256024	EU255928	KU563477
KP163290	JX463637	FJ958768	EU256025	EU255927	KU563476
KP163289	JX463636	FJ958769	EU256026	EU660385	KU563475
KP163288	JX463635	FJ958770	EU256027	EU660384	KU563474

KP163287	JX463634	FJ958771	EU256028	EU660383	KU563473
KP163284	JX463633	FJ958773	EU256029	EU595699	KU563472
KP163277	JX463631	FJ958774	EU256030	EU595698	KU563471
KP163276	JX463629	FJ958775	EU256031	EU595697	KU563469
KP163275	JX463627	FJ958776	EU256032	EU569723	KU563468
KP163274	JX463626	FJ958777	EU256033	EU569722	KU563467
KP163273	JX463625	FJ958778	EU256034	EU529681	KU563466
KP163271	JX463621	FJ958779	EU256035	EU529680	KU563465
KP163267	JX463619	FJ958780	EU256036	EU529679	KU563462
KP163264	JX463617	FJ958781	EU256037	EU529678	KU563461
KP163256	JX463615	FJ958782	EU256038	EU529677	KU563460
KP163255	JX463614	FJ958783	EU256039	EU529676	KU563459
KP163253	JX463612	FJ958785	EU256040	EU234065	KU563456
KP163249	JX463610	FJ958786	EU256041	EU155380	KU563454
KP163248	JX463609	FJ958788	EU256043	EU155378	KU563453
KP163247	JX463607	FJ958790	EU256044	EU155355	KU563448
KP163244	JX463603	FJ958791	EU256046	EU155354	KU563447
KP163242	JX463601	FJ958792	EU256047	EU155353	KU563444
KP163240	JX463598	FJ958793	EU256048	EU155352	KU563443
KP163238	JX463595	FJ958794	EU256049	EU155351	KU563442
KP163236	JX463594	FJ958795	EU256050	EU155349	KU563441
KP163227	JX463593	FJ958796	EU256051	EU155348	KU563439
KP163222	JX463592	FJ958798	EU256052	EU155346	KU563433
KP163219	JX463590	FJ958800	EU256053	EU155344	KU563432
KP163208	JX463588	FJ958803	EU256057	EU155343	KU563431
KP163203	JX463587	FJ958804	EU256058	EU155342	KU563430
KP163202	JX463586	FJ958805	EU256069	EU155341	KU563424
KP163196	JX463584	FJ958807	EU256070	EU155340	KU563422
KP163195	JX463583	FJ958808	EU256071	EU155339	KU563421
KP163182	JX463581	FJ958809	EU256072	EU155338	KU563419
KP163181	JX463580	FJ958810	EU256073	EU155323	KU563417
KP163180	JX463579	FJ958811	EU256074	EU155322	KU563416
KP163177	JX463578	FJ958812	EU255963	EU155321	KU563415
KP163174	JX463577	FJ958815	EU255964	EU155319	KU563410
KP163173	JX463575	FJ958819	EU255965	EU155314	KU563409
KP163170	JX463573	FJ958820	EU255966	EU155313	KU563408
KP163168	JX463572	FJ958822	EU255968	EU155311	KU563407
KP163167	JX463571	FJ958823	EU255969	EU155310	KU563406
KP163166	JX463569	FJ958824	EU255970	EU155309	KU563405
KP163164	JX463567	FJ958826	EU255971	EU155299	KU563404
KP163162	JX463566	FJ958827	EU255973	EU155298	KU563402
KP163156	JX463563	FJ958828	EU255974	EU155297	KU563400
KP163155	JX463562	FJ958829	EU255975	EU155296	KU563395
KP163150	JX463561	FJ958831	EU255976	EU155295	KU563393
KP163139	JX463558	FJ958832	EU255977	EU155293	KU563392
KP163137	JX463557	FJ958833	EU255978	EU155292	KU563389
KP163136	JX463556	FJ958834	EU255979	EU155291	KU563388
KP163134	JX463552	FJ958837	EU255980	EU155290	KU563386
KP163129	JX463551	FJ958838	EU255981	EU155289	KU563385
KP163126	JX463549	FJ958840	EU255982	EU155288	KU563383
KP163125	JX463546	FJ958841	EU255983	EU155287	KU563382
KP163123	JX463545	FJ958842	EU255984	EU155286	KU563381
KP163122	JX463543	FJ958843	EU255985	EU155285	KU563380
KP163119	JX463540	FJ958844	EU255986	EU155284	KU563379
KP163109	JX463539	FJ958846	EU255987	EU155283	KU563378
KP163107	JX463537	FJ958855	EU255988	EU155282	KU563377
KP163105	JX463536	FJ958936	EU255989	EU155278	KU563376
KP163103	JX463534	FJ958937	EU255990	EU155277	KU563375
KP163102	JX463532	FJ958938	EU255991	EU155276	KU563374
KP163101	JX463531	FJ958939	EU255992	EU155275	KU563373

KP163097	JX463530	FJ958941	JX472005	EU155274	KU563372
KP163093	JX463529	FJ958943	JX472006	EU155272	KU563371
KP163085	JX463527	FJ958945	JX472007	EU155271	KU563370
KP163082	JX463526	FJ958948	JX472008	EU155270	KU563369
KP163081	JX463525	FJ958957	JX472009	EU155269	KU870952
KP163079	KC844049	FJ958960	KP212019	EU155268	KU870936
KP163077	KC283194	FJ958964	KP212022	EU155267	KU870935
KP163073	JQ062147	FJ958965	KP212023	EU155266	KU870934
KP163072	JQ914273	FJ958970	KP212030	EU155252	KU870933
KP163070	JQ914272	FJ958974	KP212034	EU155251	KU870932
KP163067	JX437367	FJ959001	KP212036	EU155250	KU870931
KP163066	JX437366	FJ959020	KP212037	EU155249	KU870930
KP163063	JX437365	FJ959021	KP212038	EU155248	KU870929
KP163062	JX437364	FJ959030	KP212039	EU482884	KU870928
KP163058	JX437362	EU934905	KP212040	EU482882	KU870927
KP163057	JX437361	FJ896264	KP212041	EU482878	KX670573
KP163055	JX437360	FJ896266	KP212044	EU482876	KX670572
KP163054	JX437359	FJ896267	KP212046	EU482873	KX670569
KP163048	JX437358	FJ896268	KP212050	EU482872	KX670568
KP163044	JX437357	FJ896271	KP212053	EU482871	KX670567
KP163043	JX437356	FJ896272	KP212054	EU482870	KX670566
KP163042	JX437355	FJ896274	KP212056	EU482869	KX632242
KP163041	JX437353	FJ896275	KM587614	EU482868	KX632241
KP163040	JX437352	FJ896278	KT735178	EU482867	KX632239
KP163039	JX437351	FJ896279	KT735179	EU482866	KX632238
KP163038	JX437350	FJ896285	KT735181	EU482865	KX632236
KP163035	JX437349	FJ896288	KT735186	EU482864	KX618482
KP163034	JX437348	FJ896291	KT735187	EU482863	KX618481
KP163031	JX437347	FJ896293	KJ437300	EU482862	KX618479
KP163029	JX437346	FJ896295	KT734522	EU482861	KX618478
KP163023	JX437345	FJ896297	KU871300	EU482858	KX618477
KP163017	JX437344	FJ896298	KU871301	EU482857	KY386886
KP163015	JX437342	FJ896300	KU871302	EU482856	KY386885
KP163014	JX437341	FJ896301	KU871303	EU482855	KY386882
KP163012	JX437340	FJ896302	KU871304	EU482854	KY386880
KP163004	JX437339	FJ896303	KU871305	EU482853	KY386879
KP162999	JX437338	FJ896305	KU871306	EU482852	KY386878
KP162998	JX437336	FJ896306	KM246376	EU482850	KY386877
KP162994	JX437335	FJ896308	KM246377	EU482848	LC109261
KP162992	JX437333	FJ896310	KT721916	EU482847	LC109226
KP162989	JX437332	FJ896311	KT721927	EU482846	LC109218
KP162980	JX437331	FJ896312	KT721930	EU482845	LC109194
KP162969	JX437330	FJ896313	KT721934	EU482844	LC109188
KP162966	JX437329	FJ896314	KT721935	EU482843	LC109187
KP162962	JX437328	FJ896316	KT721936	EU482842	LC109184
KP162958	JX437324	FJ896318	KT721937	EU482841	LC109171
KP162952	JX437323	FJ896319	KT721938	EU482840	LC109164
KP162950	JX437320	FJ896321	KT721939	EU482838	LC109115
KP162947	JX437319	FJ896323	KT721940	EU482837	LC109150
KP162945	JX437318	FJ896327	KT721941	EU482836	LC109142
KP162943	JX437317	FJ896329	KT721942	EU482835	LC109139
KP162938	JX437316	FJ896330	KT721943	EU482834	LC109127
KP162936	JX437315	FJ896331	KT721944	EU482832	KX784129
KP162933	JX437314	FJ896332	KT721945	EU482831	KX784128
KP162931	JX437313	FJ896333	KT721946	EU155246	KX784127
KP162929	JX437312	FJ896335	KT721947	EU155245	KX784126
KP162927	JX437311	FJ896337	KT721949	EU155243	KX784124
KP162924	JX437310	FJ896338	KT721959	EU155242	KX784122
KP162921	JX437308	FJ896339	KT721960	EU155240	KX784121
KP162919	JX437307	FJ896341	KT721961	EU155239	KX784120

KP162915	JX437305	FJ896344	KT721962	EU155238	KX784119
KP162914	JX437304	FJ896345	KT721967	EU155237	KX784118
KP162909	JX437302	FJ896347	KT721968	EU155236	KX784117
KP162905	JX437301	FJ896348	KT721969	EU155233	KX784115
KP162897	JX437300	FJ896349	KT721970	EU155216	KX784112
KP162894	JX437299	FJ896352	KT721971	EU155215	KX784111
KP162892	JX437298	FJ896354	KT721972	EU155214	KX784110
KP162890	JX437295	FJ896355	KT721973	EU155213	KX784109
KP162878	JX437294	FJ896356	KT721974	EU234064	KX784108
KP162876	JX437293	FJ896361	KT721979	EU234063	KX784107
KP162874	JX437292	FJ896363	KT721984	EU260396	KX784106
KP162870	JX437291	FJ896366	KT721987	EU250017	KX784105
KP162869	JX437290	FJ896367	KT721988	EU239716	KX784104
KP162867	JX437289	GQ913865	KT721989	EU239715	KX784103
KP162864	JX437287	GQ913866	KT721991	EU239713	KX784102
KP162863	JX437286	GQ913867	KT721992	EU260395	KX784101
KP162860	JX437285	HM042038	KT721995	EU155345	KX784100
KP162855	JX437284	HM042039	KT721996	EU155294	KX784099
KP162853	JX437283	HM042040	KT721997	JX463641	KX784098
KP162852	JX437282	HM042041	KT721998	JX463640	KX784097
KP162849	AB709477	HM042042	KT721999	JX463639	KX784096
KP162847	HM000539	HM042043	KT722000		
KP162841	HM000536	HM042044	KT722001		
KP162840	HM000530	HM042045	KT722002		
KP162839	HM000528	HM042046	KT722003		
KP162838	HM000527	HM042047	KT722004		
KP162836	HM000526	HM042048	KT722005		
KP162831	HM000525	HM042049	KT722006		
KP162830	HM000524	JX112366	KT722011		
KP162826	HM000522	JX112367	KT722012		
KP162824	HM000518	JX112369	KT722019		
KP162821	HM000517	JX112371	KT722020		
KP162820	HM000516	JX112381	KT722021		
KP162819	HM000515	JX112387	KT722024		
KP162818	HM000514	JX112388	KT722026		
KP162816	EU716392	JX112389	KT722033		
KP162815	GU117916	JX112390	KT722039		
KP162812	GQ500983	JX112392	KT722040		
KP162809	GQ500982	JX112393	KT722041		
KP162805	GQ500980	JX112394	KT722042		
KP162801	GQ500979	JX112395	KT722043		
KP162793	GQ500976	JX112396	KT722044		
KP162792	AY956469	JX112397	KT722045		
KP162791	AY956468	JX112401	KT722046		
KP162782	AY956466	JX112403	KT722047		
KP162780	AY956465	JX112409	KT722048		
KP162776	KU725878	JX112410	KT722055		
KP162774	KU725877	JX112436	KT722056		
KP162771	KU725876	JX112437	KT722057		
KP162767	KU725875	JX112439	KT722058		
KP162762	MF137058	JX112440	KT722059		
KP162757	MF137056	JX112441	KT722060		
KP162756	MF137053	JX112449	KT722061		
KP162750	MF137052	JX112451	KT722062		
KP162749	MF137050	JX112457	KT722063		
KP162745	MF137049	JX112483	KT722064		
KP162742	MF137048	JX112484	KT722065		
KP162740	MF137047	JX112485	KT722066		
KP162738	MF137045	JX112486	KT722067		
KP162734	MF137044	JX112488	KT722070		

KP162732	MF137043	JX112489	KT722071		
KP162723	MF137042	JX112490	KT722072		
KP162718	MF137041	JX112491	KT722073		
KP162716	MF137040	JX112492	KT722074		
KP162713	MF137039	JX112494	KT722075		
KP162711	MF137038	JX112498	KT722077		
KP162710	MF137037	JX112502	KT722079		
KP162704	MF137034	JX112503	KT722080		
KP162701	MF137033	JX112505	KT722081		
KP162698	MF137032	JX112507	KT722082		
KP162697	MF137031	JX112508	KT722083		
KP162693	MF137027	JX112509	KT722084		
KP162690	MF137026	JX112512	KT722085		
KP162681	MF137025	JX112513	KT722086		
KP162674	MF137024	JX112516	KT722087		
KP162666	MF137022	JX112518	KT722088		
KP162661	MF137021	JX112519	KT722089		
KP162655	MF137020	JX112526	KT722090		
KP162651	MF137018	JX112529	KT722091		
KP162650	MF137017	JX112541	KT722096		
KP162648	MF137016	JX112575	KT722107		
KP162643	MF137015	JX112576	KT722110		
KP162642	MF137014	JX112577	KT722111		
KP162630	MF137013	JX112578	KT722112		
KP162629	MF137012	JX112579	KT722113		
KP162628	MF137011	JX112580	KT722114		
KP162626	MF137010	JX112581	KT722115		
KP162616	MF137008	JX112582	KT722116		
KP162615	MF137006	JX112583	KT722117		
KP162613	MF137005	JX112584	KT722118		
KP162605	MF137004	JX112585	KT722119		
KP162602	MF137003	JX112586	KT722120		
KP162601	MF137002	JX112587	KT722121		
KP162599	MF136998	JX112588	KT722123		
KP162597	MF136996	JX112589	KT722134		
KP162594	MF136995	JX112590	KT722138		
KP162591	MF136994	JX112591	KT722147		
KP162588	MF136993	JX112592	KT722158		
KP162587	MF136989	JX112593	KT722166		
KP162582	MF136988	JX112594	KT722176		
KP162580	MF136986	JX112595	KP212024		
KP162574	MF136985	JX112597	KP212031		
KP162568	MF136983	JX112598	KP212028		
KP162565	MF136982	JX112599	KP212032		
KP162564	MF136981	JX112601	KP212029		
KP162561	MF136980	JX112602	KP212035		
KP162558	MF136979	JX112603	KP212045		
KP162552	MF136978	JX112604	KP212042		
KP162551	MF136975	JQ914271	KP212048		
KP162546	MF136973	JQ914273	KP212057		
KP162542	MF136972	JQ061380	KP212052		
KP162541	MF136971	JQ061381	KP212021		
KP162540	MF136970	JQ061382	KP212020		
KP162537	MF136969	JQ061384	KP212025		
KP162535	MF136968	JQ061385	KP212026		
KP162533	MF136967	JQ061877	KP212027		
KP162527	MF136966	JQ061907	KP212033		
KP162526	MF136965	JQ062087	KP212043		
KP162525	MF136964	JQ062089	KP212047		
KP162524	MF136963	JQ062094	KP212049		

KP162522	MF136962	JQ062159	KP212051		
KP162511	MF136961	JQ062279	KX688385		
KP162502	MF136958	JQ062281	KX688384		
KP162493	MF136957	JQ062282	KX688383		
KP162492	MF136956	JQ062284	KX688382		
KP162491	MF136955	JQ061341	KX688381		
KP162488	MF136952	JQ061342	KX688380		
KP162486	MF136951	JQ061343	KX688379		
KP162485	MF136950	JQ061344	KX688378		
KP162482	MF136948	JQ061346	KX688377		
KP162481	MF136947	JQ061767	KX688376		
KP162479	MF136945	JQ061779	KX688375		
KP162478	MF136944	JQ061780	KX688374		
KP162475	MF136943	JQ061781	KX688373		
KP162474	MF136941	JQ061782	KX688372		
KP162473	MF136939	JQ061784	KX688371		
KP162470	MF136938	JQ061786	KX688370		
KP162461	MF136937	JQ061866	KX688369		
KP162458	MF136935	JQ061873	KX688368		
KP162456	MF136934	JQ061874	KX688367		
KP162455	MF136933	JQ061878	KX688366		
KP162452	MF136932	JQ061879	KX688365		
KP162447	MF136929	JQ061904	KX688364		
KP162435	MF136928	JQ061905	KX688363		
KP162434	MF136926	JQ061950	KX688362		
KP162433	MF136925	JQ061951	KX688361		
KP162431	MF136924	JQ061952	KX688360		
KP162426	MF136923	JQ061953	KX688359		
KP162424	MF136921	JQ061954	KX688358		
KP162412	MF136920	JQ061955	KX688357		
KP162410	MF136918	JQ062016	KX688356		
KP162409	KX825942	JQ062017	KX688355		
KP162406	KX825934	JQ062018	KX688354		
KP162405	KX825933	JQ062021	KX688353		
KP162402	KX825932	JQ062022	KX688328		
KP162400	KX825931	JQ062084	KX688327		
KP162398	KX825930	JQ062085	KX688326		
KP162397	KX825923	JQ062088	KX688325		
KP162396	KX825922	JQ062091			
KP162395	KX825921	JQ062151			
KP162394	KX852154	JQ062152			
KP162393	KX852155	JQ062153			
KP162392	KX852166	JQ062154			
KP162387	KX852168				
KP162383	KX852169				
KP162381	KX852170				
KP162380	KX852171				
KP162377	KX852172				
KP162376	KX852173				
KP162373	KP162356				
KP162372	KP162346				
KP162368	KP162345				
KP162367	KP162363				
KP162365					

Only public sequences gathered from Genbank are listed in this table, excluding the newly generated sequences from Spain.
